# Histone H1.2 Inhibited EMCV Replication through Enhancing MDA5-Mediated IFN-β Signaling Pathway

**DOI:** 10.3390/v16020174

**Published:** 2024-01-24

**Authors:** Yangran Song, Huixia Li, Ruiya Lian, Xueer Dou, Shasha Li, Jingying Xie, Xiangrong Li, Ruofei Feng, Zhiqiang Li

**Affiliations:** 1 Key Laboratory of Biotechnology and Bioengineering of State Ethnic Affairs Commission, Biomedical Research Center, Northwest Minzu University, Lanzhou 730030, China; 13033906202@163.com (Y.S.); lihui.xia@163.com (H.L.); lianruiya@163.com (R.L.); douxueer12@163.com (X.D.); lishsh91@163.com (S.L.); xjy_1314@126.com (J.X.); lixiangrong@xbmu.edu.cn (X.L.); 2 College of Life Science and Engineering, Northwest Minzu University, Lanzhou 730030, China; 3 Gansu Tech Innovation Center of Animal Cell, Biomedical Research Center, Northwest Minzu University, Lanzhou 730030, China; 4 Department of Medicine, Northwest Minzu University, Lanzhou 730030, China

**Keywords:** Encephalomyocarditis virus, histone H1.2, interferon-β, MDA5, IRF3

## Abstract

Histone H1.2 is a member of the linker histone family, which plays extensive and crucial roles not only in the regulation of chromatin dynamics, cell cycle, and cell apoptosis, but also in viral diseases and innate immunity response. Recently, it was discovered that H1.2 regulates interferon-β and inhibits influenza virus replication, whereas its role in other viral infections is poorly reported. Here, we first found the up-regulation of H1.2 during Encephalomyocarditis virus (EMCV) infection, implying that H1.2 was involved in EMCV infection. Overexpression of H1.2 inhibited EMCV proliferation, whereas knockdown of H1.2 showed a significant promotion of virus infection in HEK293T cells. Moreover, we demonstrated that overexpression of H1.2 remarkably enhanced the production of EMCV-induced type I interferon, which may be the crucial factor for H1.2 proliferation–inhibitory effects. We further found that H1.2 up-regulated the expression of the proteins of the MDA5 signaling pathway and interacted with MDA5 and IRF3 in EMCV infection. Further, we demonstrated that H1.2 facilitated EMCV-induced phosphorylation and nuclear translocation of IRF3. Briefly, our research uncovers the mechanism of H1.2 negatively regulating EMCV replication and provides new insight into antiviral targets for EMCV.

## 1. Introduction

Encephalomyocarditis virus (EMCV) is a worldwide zoonosis pathogen with a broad range of hosts, which is a naked and single-positive-strand RNA virus, assigned to the *Cardiovirus* genus in the *Picornaviridae* family [1,2]. Clinically, in addition to the typical myocarditis and encephalitis, EMCV also causes reproductive disorders and neurological diseases in several mammals [2,3,4,5]. Although EMCV does not usually cause serious clinical symptoms in humans, there is a potential threat of virulence enhancement and cross-species transmission due to its frequent application as an activator of the melanoma differentiation-associated gene 5 (MDA5) signaling pathway [6,7]. Therefore, it is of great public health significance to reveal the interaction between host proteins and viruses and elucidate the life cycle of viruses.

As a member of the *Picornaviridae* family, EMCV is primarily recognized by the pattern recognition receptor (PRRs)-MDA5, and thus can be used as an activator of the MDA5 signaling pathway [6,7] However, EMCV-encoding proteins L^pro^, 3C^pro^, 2C^pro^ and VP2^pro^ have also been reported to antagonize the innate immune response by inhibiting the RLR/MDA5 signaling pathway [8,9,10,11]. Therefore, the regulation of host proteins in EMCV infection is a key event in the process of EMCV infection. Except for these host proteins, such as Tyrosine Kinase Non-Receptor 2 (TNK2), Wiskott-Aldrich Syndrome protein Like protein (WASL), Non-Catalytic Region of Tyrosine Kinase (NCK1), the disintegrin and metalloproteinase domain-containing protein 9 (ADAM9), and Caveolin-1, directly regulating EMCV entry [12,13,14], the heat shock protein 90β (HSP90β) [15], zinc-finger FYVE domain-containing protein (ZFYVE1) [16], and members of the tripartite motif 3 (TRIM13) [17] have also been found to indirectly facilitate viral replication by targeting the innate immune signaling pathway. In addition, host proteins heat shock protein 27 (HSP27) [18] and Members of the tripartite motif 7 (TRIM7) [19] suppress viral proliferation by positively regulating the innate immune response.

Linker histone H1.2, also named HIS1H1C or H1C, participates in some cellular processes, such as genome stability, cell cycle, and apoptosis [20,21]. H1.2 is also involved in viral infection. Kristen L. Conn et al. first found that linker histones are mobilized during Herpes Simplex Virus Type 1 (HSV-1) infection and, among them, H1.2 is the most mobilized [22]. Moreover, H1.2 enhances type I interferon (IFN) production by releasing the nucleosome and promoting the binding of IRF3 to the IFN-β promoter, and thus inhibits viral infection in influenza virus-infected cells [23]. Nevertheless, the regulatory role of H1.2 in picornavirus replication has not yet been reported. In this study, we found that the protein level of H1.2 was significantly up-regulated during EMCV infection. Then, we demonstrated that the overexpression of H1.2 inhibited EMCV infection by positively regulating the EMCV-induced MDA5 signaling pathway. Our findings first revealed the antiviral mechanism of H1.2 during EMCV infection.

## 2. Materials and Methods

### 2.1. Cells and Virus

HEK293T and BHK-21 cells were cultured in DMEM medium containing 10% newborn bovine serum (Minhai Bio, Lanzhou, China). A549 cell was cultured in an F12 Nutrient Mixture supplemented with 15% newborn bovine serum. All of the cell lines were incubated using conventional methods. An EMCV PV21 strain was propagated in the BHK-21 cells, and then the clarified supernatants were also titrated on BHK-21 cells. The virus stock was preserved at −80 °C by our laboratory.

### 2.2. Mass Spectrometry (MS) Assay

BHK-21 cells were cultured to a cell density of 80–90% on 100 mm dishes. Cells were infected with EMCV at an MOI of 0.0001, and then washed with pre-chilled PBS and collected at 0 h, 1 h, 2 h, and 4 h post-inoculation (hpi). Each time point had three replicates. Subsequently, the cell precipitate was resuspended with PBS and submitted to iTRAQ^TM^ quantitative proteomics analysis by SHANGHAI LUMING BIOTECHNOLOGY CO., LTD. Shanghai, China. The original MS/MS file data were analyzed using Proteome Discoverer^TM^ 1.3 (Thermo, Waltham, MA, USA) software, and the hamster database (*Cricetidae*.fasta) from Uniprot was used. The false-positive rate of peptide identification was controlled below 1%. Additionally, the credible proteins were screened according to the criteria of peptide ≥ 1, removal of invalid values, and the existence in all three replicates. Calculation of fold change (FC) was performed with *p* values < 0.5, and only FC > 1.2 and FC < 0.83 were set as specific and significant.

### 2.3. Plasmids and Antibodies

Plasmids encoding Myc-H1.2/H1.3/H1.4/H1.5/H1t were synthesized by Genscript Co., Ltd. (Nanjing, China). The expression plasmids Flag-MDA5/MAVS/TBK1/IRF3 (5D) (the active mutant of IRF3) were all constructed and stored by our lab.

Polyclonal antibodies anti-Flag tag (20543-1-AP), anti-Myc-tag (60003-2-Ig), anti-IFIH1/MDA5 (21775-1-AP), anti-MAVS (14341-1-AP), anti-TBK1 (28397-1-AP), anti-IRF3 (11312-1-AP), and anti-HSP90 (60318-1-Ig) were purchased from Proteintech Biotechnology (Wuhan, China). Mouse monoclonal antibody for β-tubulin (TA503129) was bought from OriGene Technologies, Inc. Anti-Phospho-IRF3 (S386) recombinant rabbit monoclonal antibody (SU03-28) and monoclonal antibodies of anti-Myc tag (EM31105), H3 (M1309-1) and H1.2 (ET1706-26), were purchased from Huabio (Hangzhou, China). Peroxidase- and Cy3-Labeled antibodies of Goat Anti-Mouse IgG (H+L) and 488-Labeled Goat Anti-Rabbit IgG (H+L) were bought from Jackson ImmunoResearch Inc. (West Grove, PA, USA).

### 2.4. RNAi Assay

Transfection of HEK293T cells with siRNA was performed using Lipofectamine 2000 reagent (Invitrogen, USA). Three siRNAs targeting *H1.2* (si001: 5′-GCACUCUGGUGCAAACGAA-3′; si002: 5′-CGGCCACUGUAACCAAGAA-3′; si003: 5′-CCAAGGUUGUCAAGCCUAA-3′) and one negative control (siNC: 5′-GUUCUCCGAACGUGTCACGU-3′) were designed and synthesized by Guangzhou Ribo Biotechnology (Guangzhou, China). The interference of H1.2 was identified using western blotting.

### 2.5. EMCV Infection and Infectivity Assays

Treated or untreated cells were inoculated with the EMCV PV2 strain (0.0001 MOI) for 12 h, 24 h, and 36 h. The viral stocks and viral RNA were obtained as described previously [18]. Subsequently, the EMCV 3D gene was detected using the method of Taqman probe RT-qPCR mentioned in reference [15]. The probe and primer sequences are described in Table 1. In addition, the viral titers were determined using Karber’s method and exhibited as 50% tissue culture infectious dose (TCID_50_).

### 2.6. RNA Extraction and RT-qPCR

The extraction of total RNA was performed using traditional methods using RNAiso Plus (9108, Takara) and then transcribed to cDNA using the PrimeScript RT reagent kit (RRO47A, TaKaRa Bio, Kusatsu, Shiga, Japan). The TB Green^®^ Premix Ex Taq™ II (Tli RNaseH Plus) (RR820A, TaKaRa Bio) was used to perform quantitative PCR (qPCR) assay. GAPDH was applied as the reference genes for normalization. The 2^−ΔΔCt^ method was used for calculating the results. The primer sequences of target genes in RT-qPCR are presented in Table 1.

### 2.7. Co-Immunoprecipitation (Co-IP) Assay

According to the instruction of Protein G Agarose (P2009) (Beyotime, Shanghai, China), Co-IP assay was carried out as follows. Briefly, the co-transfected cells in 100 mm cell culture dishes were washed once with pre-chilled PBS (pH7.0) buffer, and then lysed using 500 μL of RIPA lysis buffer (P0013B) (Beyotime, Shanghai, China) containing 1 mM phenylmethylsulfonyl fluoride (PMSF) on ice for 30 min. After centrifugation, the clarified supernatant was obtained and incubated with 2 μg specific antibodies overnight at 4 °C and then uploaded to 20 μL Protein G Agarose. Next, the mixture was centrifuged and the precipitation was washed with PBS. Finally, the immunoprecipitates were analyzed using western blotting.

### 2.8. Nuclear and Cytoplasmic Extraction

The transfected HEK293T cells with plasmids Myc-H1.2 or empty vector were mock infected or infected with EMCV (0.0001 MOI). After washing with PBS, components of nuclear and cytoplasm were separated using extracting Solution A/B/C, respectively, according to the instruction of the cell membrane/cytoplasm/nuclear protein stepwise extraction kit (BestBio, Shanghai, China). The target proteins in the components of cytoplasm and nuclei were identified with western blotting using specific antibodies.

### 2.9. Indirect Immunofluorescent Assay (IFA)

After transfection and/or infection, A549 cells cultured on slides were fixed with 4% paraformaldehyde and permeabilized with 0.25% Triton X-100, and then 5% bovine serum albumin in PBS was used to block the fixed cells. The above operations were performed at room temperature. Further, the fixed cells reacted with mouse anti-Myc tag antibodies and rabbit anti-Phospho-IRF3 (S386) antibodies for 1 h at 37 °C. After washing five times with PBS, two labeled antibodies, Cy3-conjugated Goat Anti-Mouse IgG (H+L) and 488-conjugated Goat Anti-Rabbit IgG (H+L), were mixed and added into the wells. Subsequently, the cells’ nuclei were counterstained with DAPI. Finally, cells were observed under a confocal microscope (LSM 900 with Airyscan 2).

### 2.10. Statistical Analysis

The data from RT-qPCR and Taqman probe RT-qPCR were evaluated using GraphPad Prism 9.0 (GraphPad Software, San Diego, CA, USA) using Student’s *t*-tests or one-way analysis of variance (ANOVA), which were displayed as the mean ± standard deviation (SD) of at least 3 independent experiments. *p* values are indicated using asterisks in the figures, and *p* < 0.05 is considered statistically significant.

## 3. Results

### 3.1. H1.2 Expression Inhibited EMCV Replication

**Figure 1:**
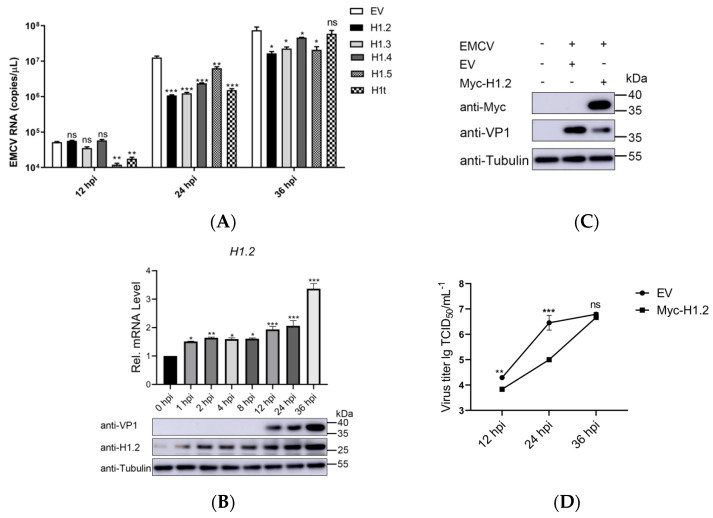
(**A**) HEK293T cells were transfected with empty vector (EV, 1 μg) or the pCMV−Myc−H1.2/H1.3/H1.4/H1.5/H1t plasmids (1 μg) for 24 h, and then were infected with EMCV at an MOI of 0.0001 for 12 h, 24 h, and 36 h. Genomic copies were determined using RT−qPCR. (**B**) HEK293T cells were infected with EMCV at an MOI of 0.0001 at 0 h, 1 h, 2 h, 4 h, 8 h, 12 h, 24 h, and 36 h. RT−qPCR (up) and immunoblotting (down) were used to detect the H1.2 expression. (**C**) HEK293T cells were transfected with empty vector (EV, 1 μg) or the pCMV−Myc−H1.2 plasmids (1 μg) and infected with EMCV at an MOI of 0.0001 at 24 h post-transfection. Immunoblotting was used to analyze the protein expression of Myc-H1.2 and VP1. Tubulin was used as a loading control. (**D**) Empty vector (EV, 1 μg) or the pCMV−Myc−H1.2 plasmids (1 μg) were transfected into HEK293T cells for 24 h and then infected with EMCV at an MOI of 0.0001 for 12 h, 24 h, or 36 h. Viral titers were detected using TCID_50_ assay (Karber’s method). Data are listed as mean ± SD of three independent experiments and measured in technical duplicate. *** *p* < 0.001; ** *p* < 0.01, * *p* < 0.05. ns means “no significant”.

Based on the proteomics analysis, 14 host proteins were identified and significantly regulated during EMCV infection (1 h, 2 h, and 4 h post-infection) (Table S1). Interestingly, among them, the 5 members of the linker histone family were significantly up-regulated by EMCV infection. To determine these linker histones’ roles in EMCV replication, we synthesized their recombinant plasmids and found that almost all of the overexpression of the 5 histones inhibited EMCV replication at 24 h postinfection (Figure 1A). Among them, the linker histone H1.2 exhibited the most significant inhibitory effect on EMCV replication. Therefore, we targeted H1.2 to explore its inhibition mechanism on EMCV replication. Furthermore, the up-regulation of H1.2 mRNA and protein level was also confirmed in HEK293T cells infected with EMCV. Compared with the control cells (0 hpi), H1.2 expression sharply increased at 12 h, 24 h, and 36 h post EMCV infection (Figure 1B). In contrast, there was no significant change in the protein abundance of H1.2 in the cells infected by UV-inactivated EMCV (with a UV dose of 100 mJ/cm^2^) (Figure S1).

To further evaluate the function of H1.2 in EMCV infection, we measured the viral protein levels of EMCV in HEK293T cells expressing Myc-H1.2. After transcription with H1.2-expressing plasmids, the cells were mock-infected or infected with EMCV for 24 h and the protein expression of EMCV VP1 was detected using western blotting. As shown in Figure 1C, the viral protein level of EMCV was inhibited significantly in HEK293T cells expressing Myc-H1.2. Moreover, the viral titers were also determined to assess EMCV yields. HEK293T cells were transfected with empty or H1.2-expressing plasmids and then infected with EMCV for 12, 24, or 36 h. The cells were collected and used for TCID_50_ assays. As shown in Figure 1D, overexpression of H1.2 significantly suppressed the viral titers of EMCV. These results demonstrated that overexpression of H1.2 down-regulated EMCV replication.

### 3.2. Knockdown of H1.2 Promoted EMCV Replication

**Figure 2:**
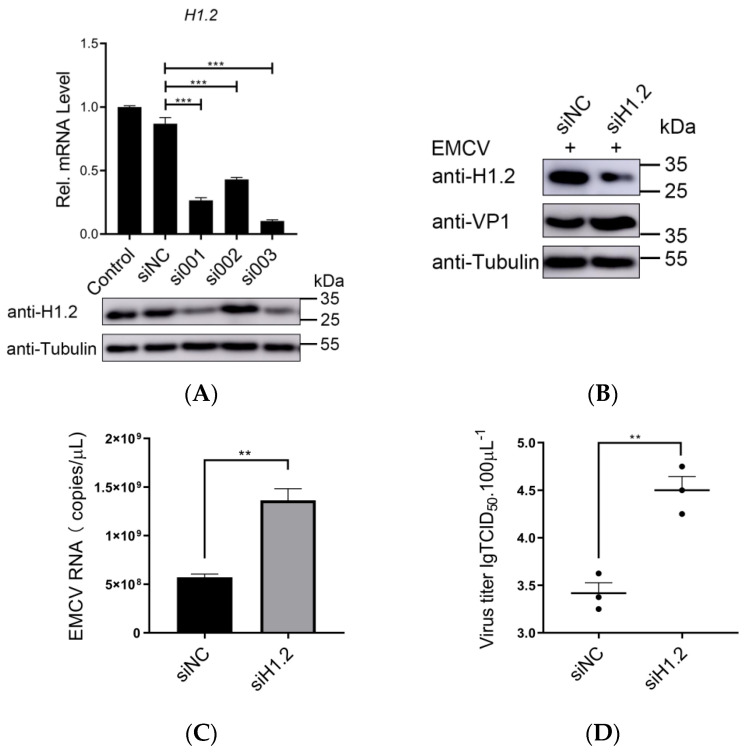
(**A**) siRNA targeting H1.2 was transfected into HEK293T cells for 24 h. Expression of H1.2 was explored with western blotting and RT−qPCR separately. *** *p* < 0.001. (**B**) siRNA targeting *H1.2* (si003) was transfected into HEK293T cells for 24 h. Cells were then infected with EMCV at an MOI of 0.0001 for 24 h. The protein expression of H1.2 and VP1 were analyzed using immunoblotting. Tubulin was used as a loading control. (**C**,**D**) HEK293T cells were transfected with siRNA targeting *H1.2* (si003) for 24 h and then infected with EMCV at an MOI of 0.0001 for 24 h. Genomic copies were determined using RT−qPCR (**C**) and viral titers were detected using TCID_50_ assay (Karber’s method). (**D**) Data are presented as mean ± SD of three independent experiments and measured in technical duplicate. ** *p* < 0.01.

To further verify the role of H1.2 in EMCV replication, small RNAi was designed and used for the knockdown of H1.2. HEK293T cells were transfected respectively with nontargeting control siRNA (siNC) or three siRNAs targeting H1.2. The expression levels of H1.2 were determined using western blotting and RT-qPCR at 24 h post-transfection. As shown in Figure 2A, expression of H1.2 was decreased remarkably in cells transfected with si001 and si003. To confirm the specificity of si003 targeting for H1.2, we also examined the expression of the other four subtypes (H1.3, H1.4, H1.5, and H1t). Transfection of siRNA (si003) had no significant effect on the expression of other subtypes (Figure S2). To determine the role of endogenous H1.2 in EMCV replication, the viral protein level, genomic copies, and viral titers of EMCV were detected in H1.2 knockdown HEK293T cells infected with EMCV. We found that the expression level of EMCV structural protein VP1 (Figure 2B), genomic copies (Figure 2C), and viral titers (Figure 2D) were up-regulated observably in H1.2 knockdown cells compared to the control cells. Therefore, H1.2 knockdown increased EMCV replication, which further confirmed the negative regulatory effect of H1.2 expression on EMCV replication.

### 3.3. H1.2 Up-Regulated IFN-β Expression Induced by EMCV

**Figure 3:**
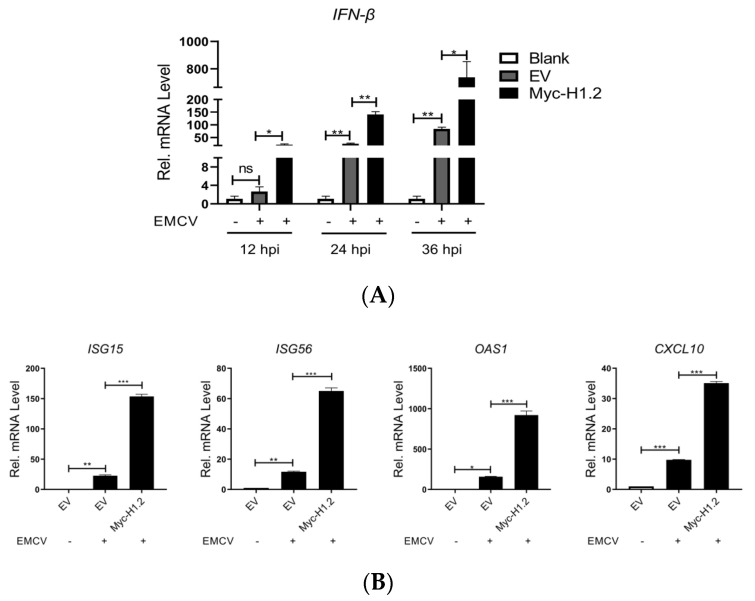
(**A**) HEK293T cells were transfected with empty vector (EV, 1 μg) or the pCMV−Myc−H1.2 plasmids (1 μg) for 24 h before being infected with EMCV at an MOI of 0.0001 for 12 h, 24 h, or 36 h. *IFN−β* mRNA expression was measured using RT-qPCR. Mock cells were used as control. ** *p* < 0.01, * *p* < 0.05 and ns means “no significant”. (**B**) HEK293T cells were transfected with empty vector (EV, 1 μg) or the pCMV−Myc−H1.2 plasmids (1 μg) for 24 h before being infected with EMCV at an MOI of 0.0001 for 24 h. *ISG15*, *ISG56*, *OAS1*, and *CXCL10* mRNA levels were measured using RT−qPCR. EV groups were used as control. *** *p* < 0.001; ** *p* < 0.01, * *p* < 0.05.

H1.2 showed an antiviral role against picornavirus EMCV. The function of H1.2 in picornavirus replication has never been reported until now. Therefore, analysis of the mechanism used by H1.2 to be involved in EMCV replication is necessary for an improved understanding of the interaction between picornavirus and host. We next investigated the role of H1.2 in host innate immune response. HEK293T cells were transfected with H1.2-expressing plasmids or empty vector plasmids and then infected with EMCV for 12, 24, or 36 h. The cells were collected for detection of IFN-β mRNA level. As shown in Figure 3A, overexpression of H1.2 promoted *IFN-β* production in EMCV infection. Moreover, the mRNA expression of *ISG15*, *ISG56*, *CXCL10*, and *OAS1* was also examined in HEK293T cells infected with EMCV. Consistently, H1.2 significantly increased the expression of *ISG15*, *ISG56*, *CXCL10*, and *OAS1* in EMCV infection (Figure 3B). This implied that H1.2 might target an EMCV-induced innate immune response to inhibit EMCV replication.

### 3.4. H1.2 Promoted EMCV-Triggered MDA5 Signaling Pathway

**Figure 4:**
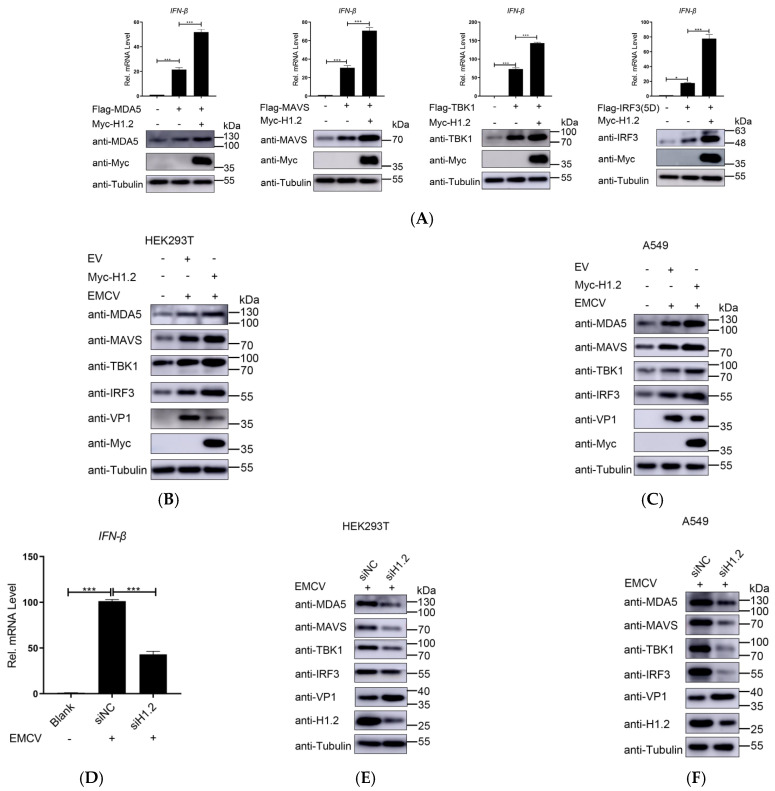
(**A**) In HEK293T cells, the empty vector (EV, 1 μg) or pCMV−Myc−H1.2 plasmids (1 μg) were co-transfected with indicated plasmids expressing MDA5 (250 ng), MAVS (250 ng), TBK1 (250 ng), or IRF3 (5D) (250 ng) for 24 h. The total RNA of cells was extracted and IFN−β mRNA levels were analyzed using RT−qPCR. GAPDH was used as a loading control. Data are presented as mean ± SD of three independent experiments, measured in technical duplicates. *** *p* < 0.001 and * *p* < 0.05. Western blotting was used to assess the expression of these signaling molecules as well as the H1.2 protein. HEK293T cells (**B**) and A549 cells (**C**) were transfected with the empty vector (EV, 1 μg) or pCMV−Myc−H1.2 plasmids (1 μg) and then infected with EMCV at an MOI of 0.0001 for 24 h. The protein levels of endogenous MDA5, MAVS, TBK1, and IRF3 were detected using western blotting. HEK293T cells were transfected with siRNA targeting H1.2 for 24 h and then infected with EMCV at an MOI of 0.0001 for 24 h. The mRNA expression of IFN−β was measured using RT−qPCR (**D**), and the endogenous proteins were detected using western blotting (**E**). A similar experiment was performed in A549 cell (**F**).

The MDA5 signaling pathway plays a critical role in sensing positive-strand picornaviruses, such as EMCV. MDA5 triggers a downstream type I IFN signaling cascade through the activity of multiple signaling proteins, including MAVS, TBK1, and IRF3. To investigate whether H1.2 regulates MDA5-mediated signaling pathways, we detected the effect of H1.2 on IFN-β expression induced by MDA5, MAVS, TBK1, or IRF3 (5D). As shown in Figure 4A, the overexpression of these signaling components significantly activated the mRNA expression of IFN-β. Moreover, the activation of IFN-β induced by these molecules was increased significantly in H1.2-expressing cells (Figure 4A). Meanwhile, we found that the protein levels of MDA5, MAVS, TBK1, and IRF3 (5D) were upregulated significantly by H1.2 compared to control groups (Figure 4A). Similarly, the protein levels of endogenous MDA5, MAVS, TBK1, and IRF3 were significantly increased by H1.2 in EMCV-infected HEK293T cells (Figure 4B) and A549 cells (Figure 4C). The results indicated that H1.2 was involved in stabilizing the expression of these four signaling molecules. To further verify the role of endogenous H1.2 in EMCV infection, H1.2 was knocked down through siRNA in HEK293T cells and A549 cells prior to EMCV infection, and mRNA expression of IFN-β was measured using RT-qPCR. As shown in Figure 4D, the knockdown of H1.2 inhibited the expression of IFN-β in HEK293T cells. Western blotting also was used to determine the abundance of MDA5, MAVS, TBK1, and IRF3 in H1.2 knockdown HEK293T cells in EMCV infection. As shown in Figure 4E, the knockdown of H1.2 expression significantly decreased the abundance of MDA5, MAVS, TBK1, and IRF3 in EMCV infection. Furthermore, the knockdown of H1.2 promoted EMCV replication due to increased VP1 protein expression (Figure 4E). Furthermore, similar results were observed in A549 cells (Figure 4F). This suggested that down-regulation of H1.2 expression impaired the EMCV-induced MDA5 signaling. Taken together, these data suggest that H1.2 positively regulated the EMCV-induced MDA5 signaling pathway by stabilizing the protein expression of MDA5, MAVS, TBK1, and IRF3.

### 3.5. H1.2 Interacted with MDA5 and IRF3

**Figure 5:**
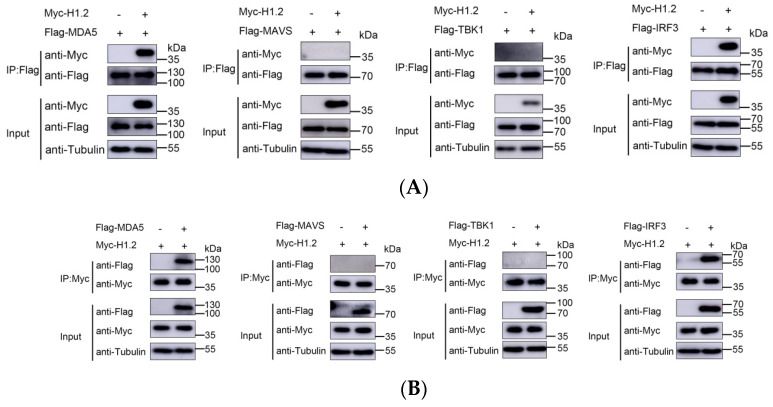
(**A**,**B**) HEK293T cells were co-transfected with pCMV−Myc−H1.2 (1 μg) and pCMV−Flag−MDA5 (1 μg), pCMV−Flag−MAVS (1 μg), pCMV−Flag−TBK1 (1 μg), or pCMV−Flag−IRF3 (1 μg) for 36 h. The cells were used for co-immunoprecipitation and immunoblotting assays with indicated antibodies.

Next, to identify whether H1.2 interacts with one or several of the MDA5 signaling pathway proteins to regulate EMCV-induced IFN-β production, the expressing plasmids of Flag-tagged MDA5, MAVS, TBK1, or IRF3, along with plasmids expressing Myc-tagged H1.2, were co-transfected into HEK293T cells. The transfected cells were subject to Co-IP assays and it was found that MDA5 and IRF3 were coimmunoprecipitated by H1.2 protein, but not MAVS and TBK1 (Figure 5A), suggesting that H1.2 both interacted with MDA5 and IRF3. Reverse Co-IP experiments using anti-Flag antibody for immunoprecipitation were also performed, and Flag-H1.2 was effectively coimmunoprecipitated by MDA5 and IRF3 (Figure 5B).

**Figure 6:**
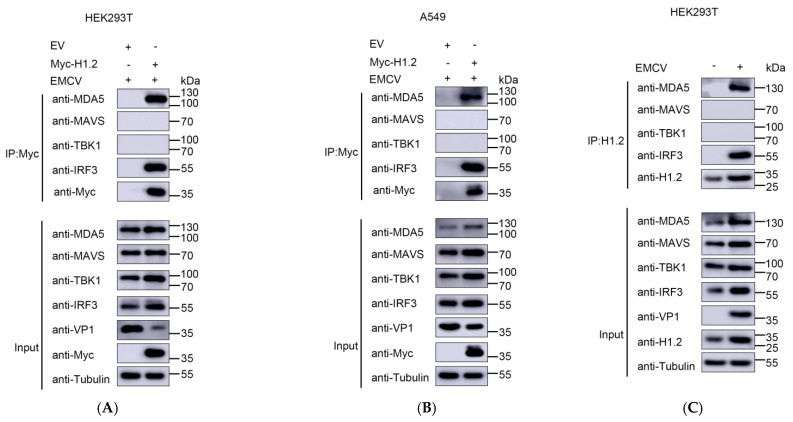
HEK293T cells (**A**) or A549 cells (**B**) were transfected with pCMV−Myc−H1.2 (1 μg) or pCMV−Myc (1 μg) for 24 h and then infected with EMCV at an MOI of 0.0001 for 24 h. The cells were collected and lysed to perform co-immunoprecipitation and western blotting assays using indicated antibodies. (**C**) HEK293T cells were mock-infected or infected with EMCV at an MOI of 0.0001 for 24 h. Cells were collected to perform co-immunoprecipitation using the antibody for H1.2. Western blotting was used to analyze the target proteins using indicated antibodies.

To further confirm the interaction of H1.2 with MDA5 and IRF3 during EMCV infection, Myc-tagged H1.2 was transfected into HEK293T cells or A549 cells and then inoculated with EMCV for 24 h. Co-IP assay was performed using an anti-Myc antibody, and western blotting analysis showed that Myc-H1.2 could coimmunoprecipitate with endogenous MDA5 and IRF3 in both EMCV-infected HEK293T (Figure 6A) and A549 cells (Figure 6B). Importantly, the interaction of endogenous H1.2 with MDA5 and IRF3 was also observed in EMCV-infected cells (Figure 6C). These results indicated that H1.2 interacted with MDA5 and IRF3 to up-regulate the EMCV-induced PRR MDA5 signaling pathway.

### 3.6. Overexpression of H1.2 Promoted IRF3 Nuclear Translocation

**Figure 7:**
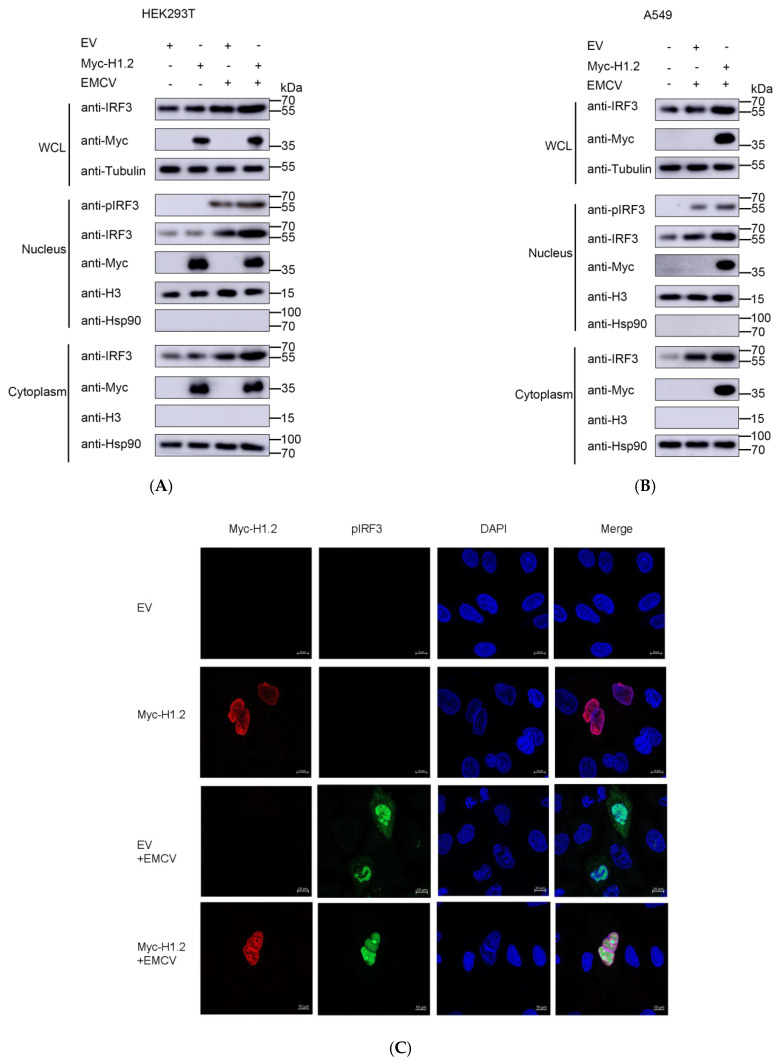
(**A**) HEK293T cells were transfected with empty vector (EV, 1 μg) or pCMV−Myc−H1.2 (1 μg) plasmids for 24 h, then cells were mock-infected or infected with EMCV at an MOI of 0.0001 for 24 h. Cells were collected and the whole-cell lysates were used for the expression analysis for IRF3. Tubulin served as a loading control. Nuclear and cytoplasmic extractions were used to detect pIRF3 and IRF3 expression in the cytoplasm and nucleus using western blotting using specific antibodies. Hsp90 was used as a loading control in cytoplasm and Histone 3 was used as a loading control in the nucleus. (**B**) A549 cells were transfected with empty vector (EV, 1 μg) or pCMV−Myc−H1.2 (1 μg) plasmids for 24 h, then cells were mock-infected or infected with EMCV at an MOI of 0.0001 for 24 h. Nuclear and cytoplasmic extractions and western blotting were performed as above (**A**). (**C**) A549 cells transfected with pCMV−Myc−H1.2 (1 μg) or pCMV−Myc (1 μg) were infected with EMCV at an MOI of 0.0001. After 24 h, expression of pIRF3 and Myc-H1.2 was detected using IFA analysis. The cells were double immunostained for Myc−H1.2 (red) and pIRF3 (green); cell nuclei were stained with DAPI. Samples were examined under a confocal microscope (LSM 900 with Airyscan 2).

We found that H1.2 interacted with IRF3. The activity of IRF3 is essential for type Ⅰ IFN production in response to the receptor recognition of viruses. IRF3 can be phosphorylated and dimerized and then translocated into the nucleus in response to the activity of MDA5. Hence, EMCV-induced nuclear translocation of IRF3 in the HEK293T cells expressing H1.2 was determined using nucleocytoplasmic separation and indirect immunofluorescence assays. As expected, the expression of pIRF3 induced by EMCV in the nucleus was improved in the cells expressing H1.2 in contrast to the control groups, and pIRF3 was not detected in the uninfected cell controls (Figure 7A). The protein level of IRF3 was enhanced in the cells expressing H1.2 in EMCV infection, which is consistent with previous results. The upregulation of H1.2 on the expression and activation of IRF3 also was found in A549 cells during EMCV infection (Figure 7B). Meanwhile, the effect of H1.2 on IRF3 nuclear translocation in EMCV infection was investigated by IFA. As shown in Figure 7C, H1.2 promoted IRF3 nuclear translocation induced by EMCV in A549 cells. Together, these findings demonstrated that H1.2 facilitated EMCV-induced phosphorylation and nuclear translocation of IRF3.

**Figure 8:**
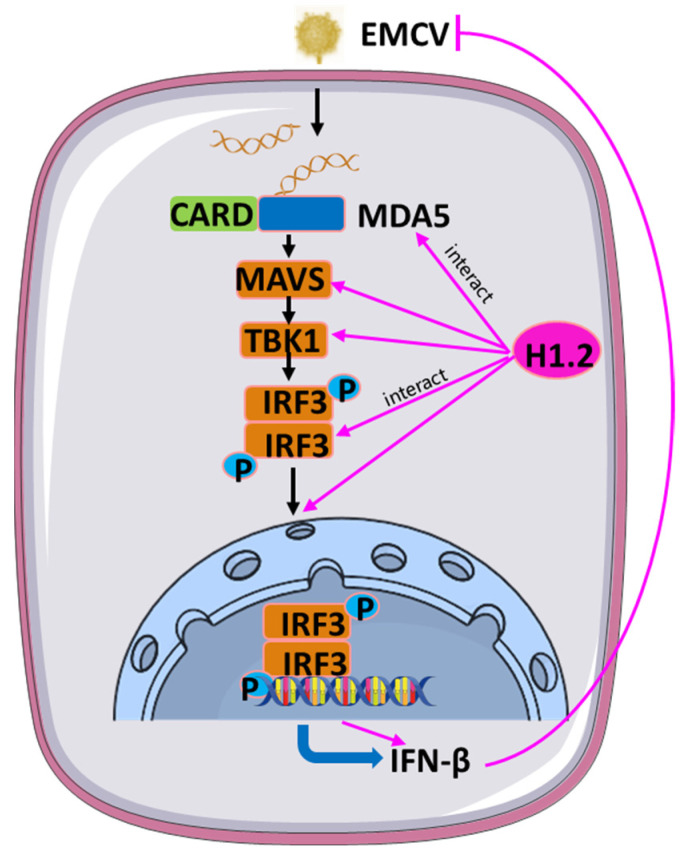
Host protein H1.2 positively regulated EMCV-induced MDA5 signaling activity by stabilizing the expression MDA5, MAVS, TBK1, and IRF3, as well as increased the phosphorylation and nuclear translocation of IRF3.

In conclusion, H1.2 plays a vital role in the EMCV-induced MDA5 signaling cascade. It positively regulated EMCV-induced MDA5 signaling activity by stabilizing the expression MDA5, MAVS, TBK1, and IRF3, as well as increased the IRF3 phosphorylation and its nuclear translocation (Figure 8).

## 4. Discussion

EMCV is exclusively prevalent in many different mammals, including domestic, captive, and wild animals via both vertical transmission and horizontal transmission [1,2,3,5]. Moreover, EMCV is clinically or subclinically circulating in humans and has a dangerous zoonotic potential for humans [24]. Therefore, the prevention and control of EMCV have important public health significance, especially in developing countries with a low level of sanitation. Uncovering the regulatory mechanism of host protein on viral proliferation is crucial to deeply understanding host–virus interaction. Here, we found that overexpression of linker histone H1.2, which is a multifunctional protein on the genome stability, cell apoptosis, cancer, and some viral infection [20], significantly inhibited EMCV replication, whereas knockdown of H1.2 facilitated EMCV viral growth. These findings imply that H1.2 may be a potential antiviral target for picornavirus.

A recent study showed that H1.2 suppresses influenza virus infection by regulating IFN-β expression [23]. Here, we first explored the roles that H1.2 play in EMCV-induced innate immune response and found that H1.2 promoted EMCV-induced IFN-β expression in HEK293T cells. Due to the critical role of the MDA5 signaling pathway in EMCV infection, we investigated and found that H1.2 enhanced the protein level of MDA5 and its down-stream signaling proteins, including MAVS, TBK1, and IRF3, during EMCV infection. Then, we preliminarily explored the mechanism of H1.2 regulating the MDA5 signaling pathway and found that H1.2 interacted with MDA5 and IRF3, as well as promoted phosphorylation and nuclear translocation of IRF3. The effect of H1.2 on IRF3 is consistent with the previous results in influenza virus infection [23]. Therefore, our study first revealed that H1.2 is involved in the regulation of picornavirus proliferation.

Reports on the relationship between histone proteins and viral infections mainly focus on the roles of core histone H2A/B, H3, and H4 or their post-translational modification (PTM) in viral infections, such as Enterovirus 71 (EV71), influenza virus, and human immunodeficiency virus (HIV) [25,26,27]. However, there are a few reports on the involvement of linker histones (H1) with viral infection. Tamura M et al. discovered the capability of Histone H1 to effectively and specifically prevent the attachment of the Norwalk virus to intestinal cells [28]. Subsequently, Conn KL et al. found that linker histones, especially H1.2, are mobilized and can regulate the expression of viral genes [22]. Regrettably, neither of them revealed the molecular mechanism of H1 regulating viral infection. Until 2017, H1.2, interacting with viral protein NS2, was known to inhibit viral replication by regulating innate immune response [23], which is consistent with our findings in EMCV infection. Mechanistically, the phosphorylation mutant (T146A) of H1.2 down-regulates IFN-β expression, while the methylation mutants (K34A, K187A) enhance IFN-β level by releasing the nucleosome and promoting IRF3 binding to the IFN-β promoter. Our study well demonstrated the antiviral effect of H1.2 in EMCV infection by targeting the MDA5 signaling pathway induced by EMCV, but future research work on exploring the roles that H1.2 post-translational modification play in EMCV infection is still worth performing.

In conclusion, we discovered that H1.2 plays a vital role in the EMCV-induced MDA5 signaling cascade. It positively regulated EMCV-induced MDA5 signaling activity by stabilizing the expression MDA5, MAVS, TBK1, and IRF3, as well as increased the phosphorylated-IRF3 and its nuclear translocation (Figure 8). Altogether, this study fills the gap of linker histones relating to picornavirus infection and provides new insight into antiviral targets for EMCV.

## Figures and Tables

**Table 1:** Primer sequences of target genes in RT-qPCR assay.

Primer Names	Primer Sequences (5′-3′)
*IFN-β* forward	TTGTTGAGAACCTCCTGGCT
*IFN-β* reverse	TGACTATGGTCCAGGCACAG
*ISG15* forward	GCAGACTGTAGACACGCTTAA
*ISG15* reverse	TTCCAATGCTATCCCAAA
*ISG56* forward	TCATCAGGTCAAGGATAGTC
*ISG56* reverse	CCACACTGTATTTGGTGTCTAGG
*OAS1* forward	ATGCTTTAAGTACAGGGACG
*OAS1* reverse	CCAAGACACTCTGGGCTA
*CXCL10* forward	ATTTGCTGCCTTATCTTTCTGACTCTA
*CXCL10* reverse	TGGCCTTCGATTCTGGATTCA
EMCV-*3D* forward	GTCATACTATCGTCCAGGGACTCTAT
EMCV-*3D* reverse	CATCTGTACTCCACACTCTCGAATG
EMCV probe	CACTTCGATCACTATGCTTGCCGTT
*GAPDH* forward	GTCTCCTCTGACTTCAACAGCG
*GAPDH* reverse	ACCACCCTGTTGCTGTAGCCAA
*H1.2* forward	CAACTCCGAAGAAGAGCGCTAAG
*H1.2* reverse	GCGTTCGCCTATTTCTTGG
*H1.3* forward	CTGGGAAACGCAAAGCATCC
*H1.3* reverse	GAAGCCGCTTTCTTGTTGAGTTT
*H1.4* forward	GAGAAGAACAACAGCCGCATCAA
*H1.4* reverse	GGTCTTCTTGGCGCTCTTCTT
*H1.5* forward	GTGAAGAAGACTCCGAAGAAGGC
*H1.5* reverse	GCAGGACTCTTGGTTGCCTTT
*H1t* forward	CCTCTCTGTGTCCAAGTTGATCA
*H1t* reverse	CACTAAGCTCTTGAGGGACAGTT

## Data Availability

All available data are presented in the article.

## Supplementary Materials

The following supporting information can be downloaded at: https://www.mdpi.com/article/10.3390/v16020174/s1, Figure S1: The effect of EMCV infection on H1.2 expression; Figure S2: Over expression or downregulation of H1.2 effect on the 5 members of H1 family. Table S1: Fourteen proteins significantly regulated during EMCV infection were identified by MS.
