# Supplementary material for: Histone H1.2 Inhibited EMCV Replication through Enhancing MDA5-Mediated IFN-β Signaling Pathway
Source: Viruses. 2024 Jan 24;16(2):174. doi: 10.3390/v16020174 (PMC10892618; doi:10.3390/v16020174)
Supplement: Supplementary file 1 [file viruses-16-00174-s001.zip › viruses-2799705-supplementary.pdf]

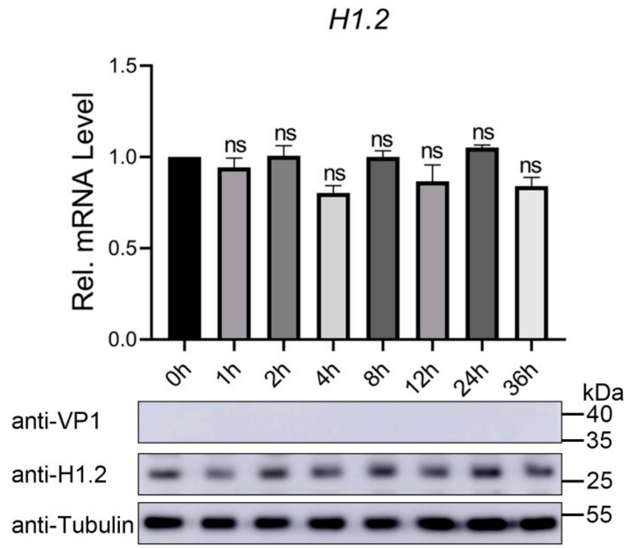

Figure S1. The effect of EMCV infection on H1.2 expression. HEK293T cells were infected with inactivated EMCV (0.0001 MOI) by a UV dose of 100 mJ/cm2 at 0 h, 1 h, 2 h, 4 h, 8 h, 12 h, 24 h and 36 h. RT−qPCR (up) and Immunoblotting (down) were used to detect the H1.2 expression. Data were listed as mean ± SD of three independent experiments and measured in technical duplicate.

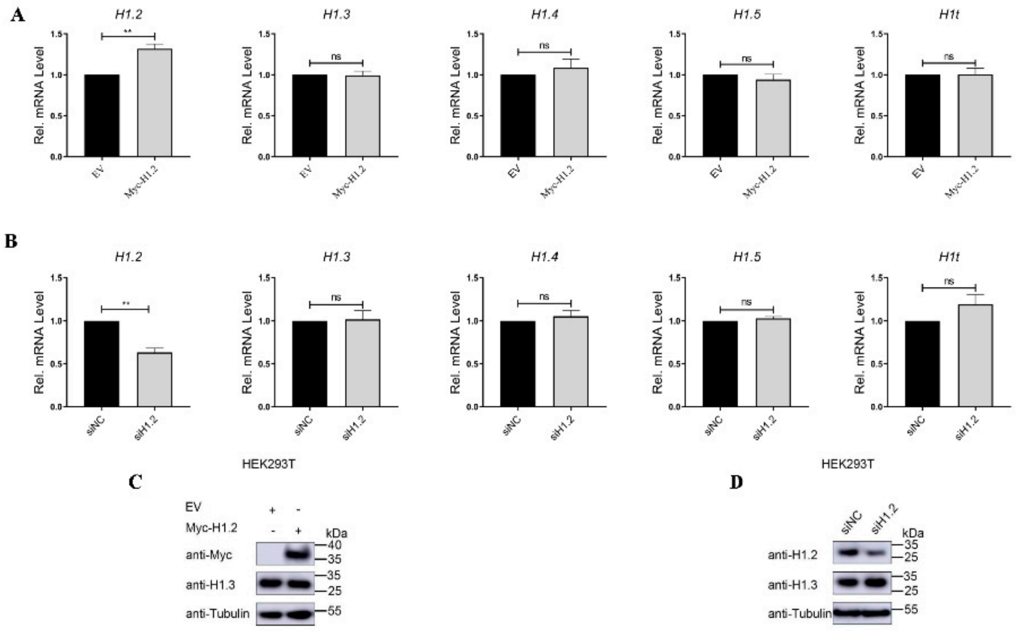

Figure S2. Over expression or downregulation of H1.2 effect on the 5 members of H1 family. (A) HEK293T cells were transfected with empty vector (EV, 1 μg) or the pCMV−Myc−H1.2 plasmids (1 μg). H1.2, H1.3, H1.4, H1.5 and H1t mRNA levels were measured by RT−qPCR. Empty vector (EV) groups were used as a control. (B) HEK293T cells were transfected with siRNA targeting H1.2 (si003). H1.2, H1.3, H1.4, H1.5 and H1t mRNA levels were measured by RT−qPCR. siNC groups were used as control. (C) HEK293T cells were transfected with empty vector (EV, 1 μg) or the pCMV−Myc−H1.2 plasmids (1 μg). Immunoblotting was used to analyze the protein expression of Myc-H1.2 and H1.3. Tubulin was used as a loading control. (D) siRNA targeting H1.2 (si003) was transfected into HEK293T cells for 24 h. The protein expression of H1.2 and H1.3 were analyzed by immunoblotting. Tubulin was used as a loading control. Data were listed as mean ± SD of three independent experiments and measured in technical duplicate. ** $p < 0.01$.

Table S1 Fourteen proteins significantly regulated during EMCV infection were identified by MS

No.	Accession	Description	Coverage	Number of AAs	MW in kDa	Number of Peptides Sequest HT	Number of PSMs Sequest HT	FC(1h-0h)	FC(2h-0h)	FC(4h-0h)
1	A0A1U8CAE9	LOW QUALITY PROTEIN: voltage-dependent anion-selective channel protein 1-like	15.7142857	280	30.204	3	19	1.48954	1.73415	1.71262
2	A0A1U8BR66	histone H1.3 OS=Mesocricetus auratus	17.7272727	220	21.963	4	126	1.84006	2.23189	2.12712
3	A0A1U8D0V7	cAMP-dependent protein kinase catalytic subunit alpha	14.8997135	349	40.181	3	9	0.81528	0.74854	0.7927
4	A0A1U8CQH7	hydroxymethylglutaryl-CoA synthase, cytoplasmic	14.2307692	520	57.386	8	21	0.74812	0.82263	0.82108
5	A0A1U7Q8Z0	histone H1.4 OS=Mesocricetus auratus	17.8082192	219	21.882	4	126	1.84006	2.23189	2.12712
6	A0A1U7QJZ4	non-histone chromosomal protein HMG-14	31.9587629	97	10.169	1	9	1.29871	1.38178	1.47638
7	A0A1U8BSH4	histone H1.5-like	18.4210526	152	16.286	3	33	1.49008	1.70657	1.66931
**8**	**A0A1U7Q3S0**	**histone H1.2**	**18.4834123**	**211**	**21.155**	**4**	**53**	**1.49392**	**1.76109**	**1.69148**
9	A0A1U8BYF8	cytochrome c oxidase subunit 4 isoform 1, mitochondrial	26.6272189	169	19.623	4	38	1.20354	1.35715	1.32123
10	A0A1U7RDX1	histone H1.5	17.6470588	221	22.434	4	53	1.49392	1.76109	1.69148
11	A0A1U7QCZ4	transmembrane protein 43	18.8679245	265	29.348	3	20	1.33345	1.57364	1.58108
12	A0A1U7QFH8	histone H1t	5.28846154	208	21.711	1	91	1.97067	2.36031	2.22152
13	A0A1U7QK94	cytochrome c oxidase subunit 5A, mitochondrial	18.0451128	133	14.612	2	18	1.24061	1.40206	1.29094
14	A0A1U7Q2N9	NADH dehydrogenase [ubiquinone] 1 beta subcomplex subunit 8, mitochondrial	26.8817204	186	21.516	4	6	1.33653	1.42121	1.45029
